# DEPRESSION AND MEDICAL COST OF CARDIOVASCULAR DISEASES

**DOI:** 10.1093/geroni/igz038.1841

**Published:** 2019-11-08

**Authors:** Guijing Wang, Chanhyun Park, Heesoo Joo, Nikki Hawkins, Jing Fang

**Affiliations:** 1 CDC, Atlanta, Georgia, United States; 2 Northeastern University, Boston, Massachusetts, United States

## Abstract

Prevalence of cardiovascular disease (CVD), the leading cause of death worldwide, increases with age. Depression is a prevalent comorbidity with CVD. This study investigates the medical costs of CVD associated with depression using a nationally representative data, 2015 Medical Expenditure Panel Survey. Patients aged ≥18 were identified by using the International Classification of Disease, 9th Revision codes of 390-459 for CVD and 296 or 311 for depression (N=23,755). Medical costs were actual payments received by providers and classified by service types and payment sources. We estimated the medical costs for each service type and payment source using economic modelling techniques controlling for various potential confounders. Overall prevalence of depression was 11.4%; 17.0% in persons with CVD and 8.7% in persons without CVD (p<0.001). Medical cost with depression was estimated at $6900 (p<0.001) for persons with CVD and $2211 (p<0.001) for those without. Costs on depression-related prescription medicines accounted for the largest portion of medical costs among persons with CVD ($3095, p<0.001). For persons with depression but without CVD, costs on outpatient visits accounted for the largest proportion ($1179, p<0.001). Medicare payments accounted for the largest portion of the depression-associated costs at $3338 (p=0.014) for persons with CVD. Compared with persons without CVD, those with CVD demonstrated doubled rates of depression. Depression-associated medical costs among individuals with CVD were tripled what they were for persons without CVD. Increased costs associated with depression were mainly for prescribed medicines and were financed by Medicare programs for persons with CVD.

